# Artificial Intelligence in the Fight Against COVID-19: Scoping Review

**DOI:** 10.2196/20756

**Published:** 2020-12-15

**Authors:** Alaa Abd-Alrazaq, Mohannad Alajlani, Dari Alhuwail, Jens Schneider, Saif Al-Kuwari, Zubair Shah, Mounir Hamdi, Mowafa Househ

**Affiliations:** 1 Division of Information and Computing Technology, College of Science and Engineering Hamad Bin Khalifa University Qatar Foundation Doha Qatar; 2 Institute of Digital Healthcare University of Warwick Coventry United Kingdom; 3 Information Science Department College of Life Sciences Kuwait University Kuwait Kuwait

**Keywords:** artificial intelligence, machine learning, deep learning, natural language processing, coronavirus, COVID-19, 2019-nCoV, SARS-CoV-2

## Abstract

**Background:**

In December 2019, COVID-19 broke out in Wuhan, China, leading to national and international disruptions in health care, business, education, transportation, and nearly every aspect of our daily lives. Artificial intelligence (AI) has been leveraged amid the COVID-19 pandemic; however, little is known about its use for supporting public health efforts.

**Objective:**

This scoping review aims to explore how AI technology is being used during the COVID-19 pandemic, as reported in the literature. Thus, it is the first review that describes and summarizes features of the identified AI techniques and data sets used for their development and validation.

**Methods:**

A scoping review was conducted following the guidelines of PRISMA-ScR (Preferred Reporting Items for Systematic Reviews and Meta-Analyses Extension for Scoping Reviews). We searched the most commonly used electronic databases (eg, MEDLINE, EMBASE, and PsycInfo) between April 10 and 12, 2020. These terms were selected based on the target intervention (ie, AI) and the target disease (ie, COVID-19). Two reviewers independently conducted study selection and data extraction. A narrative approach was used to synthesize the extracted data.

**Results:**

We considered 82 studies out of the 435 retrieved studies. The most common use of AI was diagnosing COVID-19 cases based on various indicators. AI was also employed in drug and vaccine discovery or repurposing and for assessing their safety. Further, the included studies used AI for forecasting the epidemic development of COVID-19 and predicting its potential hosts and reservoirs. Researchers used AI for patient outcome–related tasks such as assessing the severity of COVID-19, predicting mortality risk, its associated factors, and the length of hospital stay. AI was used for infodemiology to raise awareness to use water, sanitation, and hygiene. The most prominent AI technique used was convolutional neural network, followed by support vector machine.

**Conclusions:**

The included studies showed that AI has the potential to fight against COVID-19. However, many of the proposed methods are not yet clinically accepted. Thus, the most rewarding research will be on methods promising value beyond COVID-19. More efforts are needed for developing standardized reporting protocols or guidelines for studies on AI.

## Introduction

### Background

COVID-19 broke out in Wuhan, Hubei Province, China in December 2019 [1], spreading across the world and, as of May 2020, claiming the lives of more than 330,000 people [2]. Caused by SARS-CoV-2, COVID-19 was declared a global pandemic by the World Health Organization in March 2020 [3]. Many individuals infected with COVID-19 experienced fever, dry cough, and fatigue; some faced a severe course of the medical condition, often requiring intensive care, including mechanical ventilation [4]. The contagious COVID-19 and its unprecedented volume of cases around the world have caused national and international disruptions to business, health care, education, transportation, and nearly every aspect of our daily lives [5]. Prompt and effective countermeasures are necessary to cap off the effects of this pandemic; comprehensive public health strategies that involve surveillance, diagnostics, clinical treatment, and research are required [6].

Leveraging digital tools and technologies to combat COVID-19 can augment public health strategies [7], for example, by using chatbots to address public inquiries about COVID-19. Additionally, using digital tools, public health professionals can track in real time the incidence of COVID-19 infections and potentially model their projection. Among such tools is artificial intelligence (AI)—a branch of computer science concerned with intelligently analyzing and handling complex information [8,9]—amplifying public health efforts against COVID-19. Despite the enthusiasm for AI applications since the 1950s, only recently have we witnessed interest in AI due to the availability of high-performance computing and vast amounts of data being generated every second [10].

AI enables machines to become intelligent, understand queries, sift through and connect mountains of data points, and draw actionable conclusions [11]. Although defining the taxonomy of AI is not trivial, its methods can be categorized based on the objective pursued: learn from knowledge, explore and discover knowledge, extract conclusions, and reason from knowledge [8].

Soon after the COVID-19 pandemic spread across the world, several governments, research institutes, and technology companies have issued calls to action urging researchers to develop AI applications to assist with COVID-19–related research [12]. From a hierarchical perspective, AI can support COVID-19 at different levels: the molecular level (eg, drug and vaccine discovery), patient level (eg, patient diagnosis), and population level (eg, epidemiological surveillance) [13].

A full review of the AI field is beyond the scope of this review, and we refer the reader to some surveys (eg, [14]) and lectures (eg, [15,16]). However, we provide a compact overview of the AI-based techniques occurring most frequently in included studies in Multimedia Appendix 1.

### Research Problem

AI has the ability to analyze big data sets through aggregating and sifting through mountains of health care data (including patient data) to generate insights that can enable predictive analysis. The quick ability to obtain these insights helps clinicians as well as other stakeholders in the health care ecosystem to make effective, safe, and timely decisions to better serve patients and public health policy. There has been a steady rise in the number of studies regarding the use of AI techniques to resolve or address the COVID-19 pandemic [13]. Much of the AI research effort during the COVID-19 pandemic has been scattered, and a need to explore and summarize how AI technologies are being used to resolve or address the many challenges relating to COVID-19 can help us plan on how to leverage AI technologies in the current or a future pandemic. Several reviews have been conducted on AI techniques used to address the COVID-19 pandemic [12,13,17-20]. However, much of the work has been in the form of literature reviews [12,13,17-19] or systematic reviews focusing on one application of AI (eg, diagnosis and prognosis of COVID-19) [20]. Therefore, it is necessary to conduct a more systematic and comprehensive review that focuses on all applications of AI used amid the COVID-19 pandemic. Accordingly, this review aims to explore how AI technology is being used during the COVID-19 pandemic as reported in the literature. The results can be useful for health care professionals and policy makers considering leveraging AI to complement public health efforts in response to COVID-19.

## Methods

To achieve the objective of this study while ensuring both replicable and transparent methods, we conducted a scoping review following the guidelines of PRISMA-ScR (Preferred Reporting Items for Systematic Reviews and Meta-Analyses Extension for Scoping Reviews) [21]. Methods used in this review are detailed in the following subsections.

### Search Strategy

#### Search Sources

In this review, we performed search queries between April 10 and 12, 2020, on the following online databases: MEDLINE (via Ovid), EMBASE (via Ovid), PsycInfo (via Ovid), IEEE Xplore, ACM Digital Library, arXiv, medRxiv, bioRxiv, Scopus, and Google Scholar. In the case of Google Scholar and due to the volume of returned hits, only the first 100 results were considered, as we found that, beyond this, results quickly lose relevance and applicability. In addition to searching bibliographic databases, we screened the reference list of the included studies and relevant reviews to look for other relevant studies that could be added to this review (ie, backward reference list checking).

#### Search Terms

The search terms we used to identify relevant studies were specified from the available literature and by referring to subject matter experts. These terms were selected based on the target intervention (eg, AI, machine learning, and deep learning) and the target disease (eg, coronavirus, COVID-19, and 2019-nCoV). Details about the exact search strings used in this study are provided in Multimedia Appendix 2.

### Study Eligibility Criteria

In this review, we focused on any AI-based technology or approach used for any purpose related to the COVID-19 pandemic, such as diagnosis, epidemiological predictions, treatment and vaccine discovery, and prediction of patient outcomes. However, we excluded studies providing an overview or proposing a potential AI technique for COVID-19, or studies that were purely discussed from a research perspective.

We considered studies published in English between December 25, 2019, and April 12, 2020, such as peer-reviewed articles, theses, dissertations, conference proceedings, and preprints, while excluding other publications such as reviews, conference abstracts, proposals, editorials, and commentaries. We did not enforce any restrictions on the country of publication, study design, comparator, and outcomes.

### Study Selection

Two reviewers, namely, authors AAA and MA, independently screened the titles and abstracts of the identified studies. They independently read the full text of studies that passed the *title and abstract* screening. We then investigated any disagreement between AAA and MA and resolved them through discussion and consensus. We calculated Cohen kappa [22] to measure the reviewer’s agreement and found it to be 0.83 for the *title and abstract* screening and 0.94 for the full-text reading, indicating a very good agreement [23]. Multimedia Appendix 3 shows a matrix of interrater agreement in each step.

### Data Extraction

Multimedia Appendix 4 shows a purpose-built data extraction form, which was pilot-tested using 7 relevant studies to accurately extract data. The two reviewers (AAA and MA) independently extracted data related to characteristics of the included studies, AI techniques, and data sets used for the development and validation of AI models. Like the study selection process, any disagreement between the reviewers was resolved through consensus. We calculated Cohen kappa [22] and found it to be 0.88, meaning a very good agreement [23].

### Data Synthesis

After extracting the data from the identified studies, we used a narrative approach to synthesize it. Specifically, we classified and described AI techniques used in the included studies in terms of their purposes (eg, diagnosis and drug and vaccine development), AI area or branch (eg, traditional machine learning and deep learning), AI models and algorithms (eg, decision tree, random forest, and naive Bayes), and platform (ie, computer and mobile). Further, we described the data sets used for development and validation of AI models in terms of data sources (eg, public databases and clinical settings); type of data (eg, radiology images, biological data, and laboratory data); size of the data set; type of validation; and proportion of training, validation, and test data sets. We used Microsoft Excel (Microsoft Corporation) to manage data synthesis.

## Results

### Search Results

**Figure 1:**
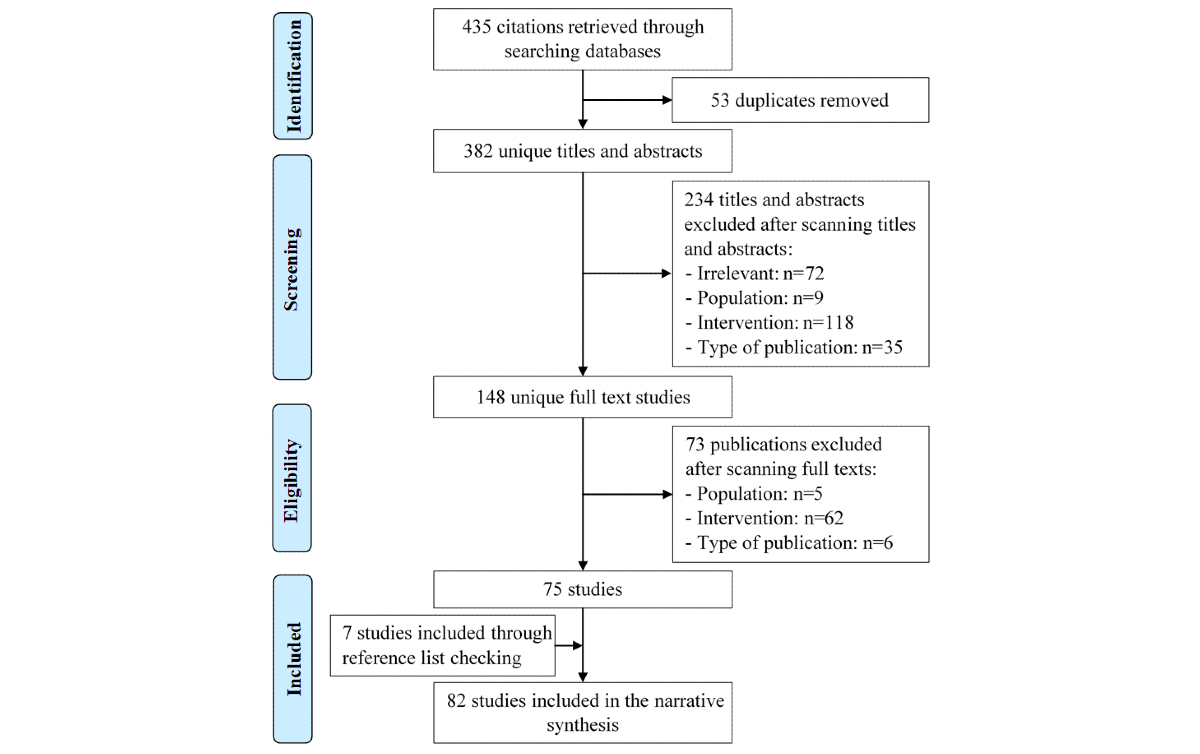
Flowchart of the study selection process.

We retrieved 435 studies through searching the identified bibliographic databases (Figure 1). Of those studies, we removed 53 duplicates; we then screened the titles and abstracts of the remaining 382 studies. The screening process led to the exclusion of 234 studies for reasons detailed in Figure 1. After reading the full texts of the remaining 148 studies, we excluded 73 studies, as they did not meet all eligibility criteria. Thus, we included the remaining 75 studies. We identified 7 additional studies by checking reference lists of the included studies and relevant literature reviews. Overall, 82 studies were included in this review.

### Characteristics of the Included Studies

**Figure 2:**
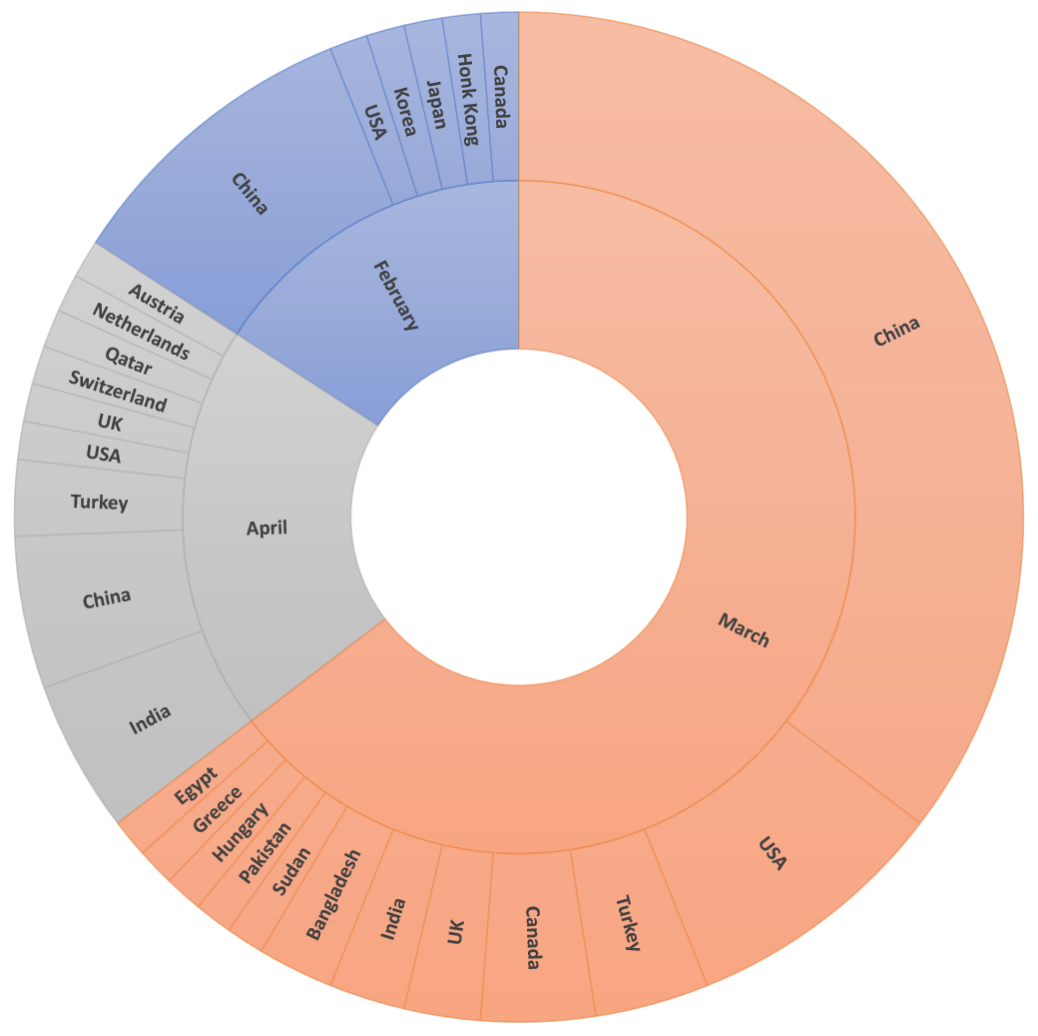
Publications by months and country.

Among the included studies, 72 were preprints and 10 were published articles in peer-reviewed journals (Table 1 and Figure 2). About two-thirds (n=53) of the included studies were submitted in March 2020, and the remaining studies were published in February and April 2020. However, no studies were published during the first 2 months of the COVID-19 outbreak. The included studies were conducted in 19 countries; however, half of the studies (n=41) were published in China. Multimedia Appendix 5 shows the characteristics of each included study.

**Table 1:** Characteristics of the included studies.

Characteristics		Studies (N=82), n
**Paper status**		
	Preprint	72
	Published	10
**Submission month**		
	February	13
	March	53
	April	16
**Country of publication**		
	China	41
	US	9
	India	6
	Turkey	5
	Canada	4
	UK	3
	Bangladesh	2
	Austria	1
	Egypt	1
	Greece	1
	Hong Kong	1
	Hungary	1
	Japan	1
	Korea	1
	Netherlands	1
	Pakistan	1
	Qatar	1
	Sudan	1
	Switzerland	1

### AI-Based Techniques Used for COVID-19

#### Purposes or Uses of AI Against COVID-19

As shown in Table 2, AI techniques have been used for five purposes amid the pandemic. In 31 studies [24-54], AI was used for diagnosing COVID-19 cases or identifying suspected COVID-19 cases based on various indicators, including computed tomography (CT) images [24-38], x-ray images [39-50], laboratory tests [51,52], genome sequences [53], and respiratory patterns [54].

**Table 2:** Purposes and uses of artificial intelligence against COVID-19.

Purposes/uses		Studies (N=82), n
**Diagnosis**		
	CT^a^ images	15
	X-ray images	12
	Laboratory tests	2
	Genome sequence	1
	Respiratory patterns	1
**Treatment and vaccines**		
	Drug discovery	9
	Vaccine discovery	4
	Protein structure	4
	Drug repurposing	2
	Treatment safety	1
**Epidemiology**		
	Epidemic development	14
	Potential reservoirs	3
**Patient outcome**		
	Severity	6
	Progression to severe	4
	Mortality risk	2
	Risk factors	1
	Hospital stay	1
**Infodemiology**		
	Raising awareness	1

^a^CT: computed tomography.

In 20 studies [55-74], AI was also harnessed for treatment and vaccines for COVID-19. Specifically, 9 studies used AI for discovering drugs suitable for combating COVID-19 [55-63], and 2 studies used AI for repurposing commercially available drugs that could be used for treating COVID-19 [64,65]. There was 1 study that employed AI to predict the safety of using traditional Chinese medicine for COVID-19 [66]. In 4 studies [67-70], AI was used for discovering COVID-19 vaccines. Another 4 studies used AI for predicting the protein structure of SARS-CoV-2, thereby aiding researchers and pharmaceutical companies to discover drugs for COVID-19 [71-74].

There were 17 studies that used AI for epidemiological modeling tasks [75-91]. In particular, 14 of these studies employed AI for forecasting the epidemic development (eg, numbers of confirmed, recovered, death, suspected, asymptomatic, and critical cases, and lengths and ending time) [75-88], and 3 studies [89-91] used AI for predicting the potential hosts or reservoirs of SARS-CoV-2.

In 14 studies [33,92-104], AI was used for patient outcome–related tasks. In particular, 6 studies used AI for segmentation and quantification of infected regions in the lungs due to COVID-19, thereby assessing the severity of the disease [92-97]. AI was also used in 4 studies for identifying cases at high risk of progression to severe COVID-19 [33,98-100]. Furthermore, AI was also used for predicting mortality risk [101,102], its associated factors [103], and the length of hospital stay in patients with COVID-19 [104].

AI has also been used for infodemiology [105]. Specifically, AI was used for raising awareness to use water, sanitation, and hygiene through combining authentic sources of information with daily news [105]. Multimedia Appendix 5 presents the purposes or uses of AI techniques in each included study.

#### Features of AI-Based Techniques Used for COVID-19

In 29 studies [24,31,47, 51,52,58, 63,68,70, 74,79,83-86,88,90,91,95-105], AI techniques used against COVID-19 were based on traditional machine learning models and algorithms (Table 3). The most commonly used machine learning models and algorithms were support vector machine (SVM) [24, 31, 47,58, 68, 70,79,91,98,101], random forest [31,58,68,74,90,96,101,103,104], decision tree [52,58,68,74,79,97,99,101,102], and logistic regression [31,51,52,58,68,99-101,104].

**Table 3:** Features of AI-based techniques used for COVID-19.

Features		Studies (N=82), n
**AI^a^ branches^b^**		
	Deep learning	60
	Machine learning	29
	Natural language processing	3
**AI models/ algorithms^c^**		
	Convolutional neural network	37
	Support vector machine	10
	Random forest	9
	Decision tree	9
	Logistic regression	9
	Recurrent neural network	8
	Artificial neural network (unspecified)	6
	Transfer learning	4
	Autoencoders	4
	Deep neural network	3
	K-nearest neighbors	3
	Least absolute shrinkage and selection operator	3
	Polynomial neural network	3
	Multilayer perceptron	2
	Advance deep Q-learning network	2
	AdaBoost	1
	Auto-regressive integrated moving average model	1
	Bayesian analysis	1
	Bidirectional encoder representations from transformers	1
	Continuous bag of words	1
	Eureqa modeling	1
	Genetic algorithm	1
	Generative adversarial network	1
	Generalized logistic growth model	1
	Holistic agent-based model	1
	Linear discriminant analysis	1
	Linear regression	1
	Language model	1
	Multi-task deep model	1
	Naive Bayes	1
	Porter stemming	1
	Reinforcement learning	1
	Skip-gram model	1
	Time series forecasting	1
	Universal-sentence-encoder-large	1
	Vector auto average	1
**Platforms**		
	Computer	81
	Mobile	1

^a^AI: artificial intelligence.

^b^Numbers do not add up as AI techniques in some studies were based on more than one AI branch.

^c^Numbers do not add up as several studies used more than one AI model or algorithm.

In 60 studies, AI techniques used against COVID-19 were based on deep learning models and algorithms [25-50,53-57,59-67,69,71-73,75-82,87,89,92-95,98,101]. The most commonly used learning models and algorithms in the included studies were convolutional neural network (CNN) [25-50,53,62,64,72,73,82,89,92-95] and recurrent neural network (RNN) [54,55,57,59,71,73,77,98].

In 2 studies [64,105], AI techniques used against COVID-19 were based on models related to natural language processing (NLP), such as the continuous bag of words model, skip-gram models, and porter stemming. Although AI techniques were implemented in mobile phones for 1 study [105], computers were the platform for AI techniques in the remaining studies. Multimedia Appendix 5 shows features of AI-based techniques used in each included study.

#### Features of Data Sets Used for Development and Validation of AI Models

As shown in Table 4, public resources (eg, National Center for Biotechnology Information [NCBI], GitHub, and Kaggle) were the most commonly used data source for development and validation of AI models [24,27, 29,36,39-50,53, 55-65,67-75,77, 80-85,87-89,91-93,103,105]. Other data sources used by the included studies were as follows: clinical settings (eg, databases in hospitals and medical centers) [25-35,37,38,51,52,63,94-98,100, 102,104], government sources (eg, Chinese Center for Disease Control and Prevention) [53,76,78, 79, 84,86, 90, 99,101], literature (eg, previous studies and books) [36,40,42,61,66,101], news websites [101,105], and participants recruited by the study [54].

**Table 4:** Features of data sets used for development and validation of artificial intelligence models.

Features		Studies (N=82), n
**Data sources^a^**		
	Public databases	52
	Clinical settings	24
	Government sources	9
	Literature	6
	News websites	2
	Participants	1
**Data types^b^**		
	Radiology image	35
	Biological data	23
	Epidemiological data	15
	Clinical data	11
	Laboratory data	8
	Demographic data	5
	Guidelines	1
	News articles	1
**Data set size^c^**		
	<1000	26
	1000-9999	16
	≥10,000	8
**Type of validation^d,e^**		
	Train-test split	25
	K-fold cross-validation	18
	External validation	11
**Proportion of training set (%)^f^**		
	≤25	3
	26-50	2
	51-75	16
	>75	28
**Proportion of validation set (%)^g^**		
	≤25	8
	26-50	3
	51-75	0
	>75	0
**Proportion of test set (%)^h^**		
	≤25	35
	26-50	10
	51-75	3
	>75	1

^a^Numbers do not add up as several studies collected their data from more than one data source.

^b^Numbers do not add up as several studies collected more than one type of data.

^c^Data set size was reported in 50 studies.

^d^Type of validation was reported in 53 studies.

^e^Numbers do not add up as 1 study used two different types of validation.

^f^Proportion of the training set was reported in 49 studies.

^g^Proportion of the validation set was reported in 11 studies.

^h^Proportion of the test set was reported in 49 studies.

The types of data collected from these data sources were as follows: radiology images (eg, CT and x-ray) [24-50,54,92-96,98,104], biological data (eg, protein and genome sequences) [53,55-65,67-74,89-91], epidemiological data (eg, number of infected and recovered cases) [75-85,87,88,97,102], clinical data (eg, signs, symptoms, physician notes, and patients’ history) [25,51,52,66,97-103], laboratory data (blood and polymerase chain reaction test results) [25,51,52,86,97,98,100,102], demographic data (eg, age, gender, and ethnicity) [52,99-102], guidelines [105], and news articles [105].

The data set size was reported by 50 studies, ranging from 31 to 3,000,000. The data set size was less than 1000 samples in half of these studies [24,27,32,34, 36,37,39, 41,44,45, 47,51-53,69,86,92,94-98,100,102-104], and only 8 studies reported a size of 10,000 samples or more [25,26,54,59,61,87,99,101].

Validation of models was reported in 53 studies. Three types of validation were used in the included studies: train-test split [25,29, 30, 34-39, 41, 43, 44, 47-50, 59, 75,87,88, 93,94,99,100,103], K-fold cross-validation [24,31,40, 42,45,46, 52,53, 58, 66,68,90-92,96,98, 101,104], and external validation [26,27,29,32,33,38,51,54,82,95,102].

The training set proportion of the total data set was reported in 49 studies. The proportion of the training set ranged from ≤25% in 3 studies [25,27,28], 26%-50% in 2 studies [26,95], 51%-75% in 16 studies [32,33,35,36, 38, 39,47, 51, 59,75,87,88,96,100,102,103], and >75% in 28 studies [24,29-31,34,37,40-46,48,50, 52-54,58, 66,90-92, 94,98,99,101,104]. The mean of the proportions of the training set in the 49 studies was 72.7%.

The validation set proportion of the total data set was reported in 11 studies; it ranged from ≤25% in 8 studies [26,28, 35, 36,38, 47, 48,53] and 26%-50% in 3 studies [25,100,102]. The mean of the validation set proportions in the 11 studies was 18.7%.

The test set proportion of the total data set was reported in 49 studies, ranging from ≤25% in 35 studies [24,29-31,34,36,37,40-48,50,52-54,58,59,66,75,87,90-92,94,98-102,104], 26%-50% in 10 studies [26,32,33,35,38,39,51,88,96,103], 51%-75% in 3 studies [25,28,95], and >75% in 1 study [27]. The mean of the test set proportions in the 49 studies was 22.9%. Multimedia Appendix 6 presents features of the data sets used for development and validation of AI models in each included study.

## Discussion

### Principal Results

In this study, we conducted a scoping review of the use of AI against COVID-19. We found a lack of publications in December 2019 and January 2020. This is not surprising, given that SARS-CoV-2 was only identified on January 7 [106]; insufficient data was not available to back scientific publications, in particular internationally; and the contagiousness and aggressiveness of the virus were underestimated (first lockdown in China was January 23 [106]). Half of the studies in this report were published in China. Since SARS-CoV-2 originated in China and affected it the most during the first 3 months of the pandemic, it had the most data related to COVID-19. Considering lengthy publication processes and the vast number of COVID-19–related manuscript submissions, it is also not surprising that most of the included studies were preprints.

In the included studies, AI was used for five purposes: diagnosis, treatment and vaccine discovery, epidemiological modeling, patient outcome–related tasks, and infodemiology. None of the included studies used AI for other purposes such as contact tracing of the individuals, providing training to students and health care professionals, or robotics to deal with suspected and quarantined cases.

Most of the AI techniques used in the included studies were based on deep learning approaches such as CNN and RNN. All but 1 study used desktop machines, workstations, and clusters as opposed to mobile platforms. This can be explained by the computational demand in training AIs. Although all major mobile phone manufacturers equip their flagship models with AI coprocessors, these coprocessors accelerate inference, a computationally much lighter task. In addition, *federated* learning [107] (a machine learning privacy-preserving technique usually used in mobile phones) is still in its infancy and raises issues such as data sovereignty, scalability, and performance.

Data sources used in the included studies usually came from the public domain (eg, NCBI, GitHub, Kaggle) and proprietary databases (less common). Radiology images were the most commonly used type of data, followed by biological data. The number of samples was still comparably small (less than 1000 in half of the studies). The diversity and size of data indicate a lack of publicly available data despite COVID-19 cases having surpassed 5 million at the time of writing. We, therefore, second Wynants et al [20] call “for immediate sharing of the individual participant data from COVID-19 studies worldwide.”

### Practical and Research Implications

Although this review explores the use of AI against COVID-19, some applications could prove useful far beyond this pandemic. For instance, Kiwibot designs autonomous medical delivery robots to minimize interpersonal contact [108]. Whiteboard Coordinator developed a high precision thermal screening device eliminating individual measurements, leading to higher throughput and larger social distances [109]. Although mobile phones are not yet the AI platform of choice, the first apps to track interpersonal contact using mobile phones have been published to prioritize COVID-19 testing [110]. Finally, whereas a real-time reverse transcription polymerase chain reaction test takes around 25 minutes and requires stocks of chemical reagents, AI can inspect chest CTs to provide preliminary diagnoses in seconds. We believe that increasing social distance and providing fully autonomous checkups will be the most valuable use of AI beyond the current pandemic.

In the past, fundamental AI research was focused mainly on faster (or even feasible) training. We believe that, in the future, this must be complemented with public education. AI mistrust, because of our still lacking understanding of how AI works at the deepest level, further raises ethical questions that need to be answered before AI will be uniformly accepted. We also found that AI features and results were reported in an inconsistent manner, potentially fueling AI mistrust and making a direct comparison between studies difficult. Of the 82 studies, we found that only 64.6% (n=53) of the studies included in this review disclosed the type of validation, 61% (n=50) mentioned the data size, and more than 7% (n=6) did not even specify the type of AI used. It is therefore important that we as a community develop a standardized reporting protocol to slow down the barrage of poorly conducted COVID-19 studies that threaten to overwhelm serious scientists (1916 related papers were retrieved before April 5, 2020 by Wynants et al [20]), strengthen properly conducted studies, and improve reproducibility.

We found that, explicably, the landscape of studies is still dominated by Chinese institutions, which bears the potential for cultural, technological, and geospatial biases. However, we see a recent move toward a more balanced landscape (see Figure 2). Although we identified more than 100 models developed in the included studies, we did not assess their quality as it was out of this review’s scope. Therefore, further reviews are needed to assess the quality of AI models used in the fight against COVID-19.

Given the current “infodemic” [13], we find it surprising that NLP is not used more often. We see AI-based analysis of effective advertisement for nonpharmaceutical interventions as one research opportunity to answer questions like what manner of speech results in maximum public acceptance.

### Strengths and Limitations

#### Strengths

Given that this review includes all AI techniques used for the COVID-19 pandemic regardless of their characteristics, study design, study setting, and country of publication, it may be considered the most comprehensive review in this research area. This helps readers to speculate how AI is being leveraged amid the COVID-19 pandemic. In comparison with similar reviews [12,13,17-20], our review is the only one that describes and summarizes features of the identified AI techniques and data sets used for their development and validation. Furthermore, unlike previous reviews [12,13,17-19], this review follows the full scientific rigor of PRISMA-ScR [21].

In contrast to other reviews, we searched the most commonly used databases in health and information technology fields to identify as many relevant studies as possible. Thus, the number of studies included in this review was much higher than in other reviews [12,13,17-20]. Additionally, we strove to retrieve gray literature and minimized the risk of publication bias by searching Google Scholar and conducting backward reference list checking. Furthermore, we minimized selection bias by having two independent reviewers conducting study selection and data extraction, with a very high agreement in both processes.

#### Limitations

Given that our review excludes proposals of AI techniques, it is likely that we missed other applications of AI for COVID-19. This review, therefore, might not identify all potential uses of AI for the current pandemic. Owing to practical constraints, the search was restricted to English studies. Therefore, we probably missed several studies written in other languages, especially Chinese. The search query did not include terms related to specific types of models or algorithms such as CNN, RNN, and SVM. Thus, it is likely that we missed some studies that used such terms in their title and abstract instead of the terms that we used (ie, AI, machine learning, and deep learning). The findings of this review are mostly based on preprints, which are more likely to have inaccurate or missing information. Therefore, the accuracy of the information in the included studies may affect the accuracy of our findings.

### Conclusions

In this study, we provide a scoping review of 82 studies on AI against COVID-19. Given that many of the proposed methods are not yet clinically accepted, we remark that the most rewarding research will be on methods promising value beyond COVID-19. We believe that mobile phones offer unexploited potential, but more research in the direction of energy-efficient and federated learning is needed. We also believe that the use of NLP to assess effective communication of nonpharmaceutical interventions is a largely unexplored research direction, especially since data driving this research is available in the public domain, unlike much of the data produced by clinical studies. For AI to gain broad acceptance, standardized reporting protocols to be followed by studies on AI are needed. Likewise, more research on AI ethics and explainable AI is needed, paired with public education initiatives.

## Appendix

Multimedia Appendix 1 Overview of artificial intelligence–based techniques.

Multimedia Appendix 2 Search strategy.

Multimedia Appendix 3 Interrater agreement matrices for study selection steps.

Multimedia Appendix 4 Data extraction form.

Multimedia Appendix 5 Characteristics of the included studies and features of artificial intelligence techniques used for COVID-19.

Multimedia Appendix 6 Features of data sets used for the development and validation of artificial intelligence models.

## Abbreviations

AI: artificial intelligence
CNN: convolutional neural network
CT: computed tomography
NCBI: National Center for Biotechnology Information
NLP: natural language processing
PRISMA-ScR: Preferred Reporting Items for Systematic Reviews and Meta-Analyses Extension for Scoping Reviews
RNN: recurrent neural network
SVM: support vector machine
