# Supplementary material for: Artificial Intelligence in the Fight Against COVID-19: Scoping Review
Source: J Med Internet Res. 2020 Dec 15;22(12):e20756. doi: 10.2196/20756 (PMC7744141; doi:10.2196/20756)
Supplement: Multimedia Appendix 1 [file jmir_v22i12e20756_app1.docx]

**Appendix 1: Overview of AI-based techniques**

In this scoping review, we broadly classify AI-based techniques in included studies into Machine Learning (“shallow learning”) techniques and Deep Learning techniques. Although Natural Language Processing (NLP) techniques can combine aspects of deep and shallow learning, we treat them as an independent class. Historically, shallow methods have first dominated AI research due to limited computational resources and understanding of multi-layer artificial neural networks (Multilayer Perceptrons, MLPs). All techniques seek to extract structure or insight from potentially large and seemingly unorganized data.

Shallow methods such as Linear Regression, Logistic Regression, LASSO, K-Nearest Neighbors, Linear Discriminant Analysis, etc. try to find clusters of data samples, either in the data’s original space (KNN) or in a lower-dimensional space. Support Vector Machines (SVMs) in contrast embed the original data in a higher-dimensional space in which finding a separating line between clusters is easier. This separating line curves under back projection to the original space and separates classes of data elements. Clusters or regions in the data domain are often labelled, and a common task is to predict the label (that is, the cluster or region) for a new, previously unseen data sample (“classification task”). Unlike deep learning techniques, shallow techniques may work well even on small data sets.

In contrast, deep methods are based on artificial neural networks, in particular multilayer perceptrons (MLPs). Here, several layers of artificial neurons are connected to form a neural architecture. By feeding large amounts of data into a numerical optimization method, a model is trained for specific tasks such as classification (prediction of labels), segmentation (finding of relevant regions), or dynamic control (recommending actions). Convolutional neural networks (CNN) have received much research interest, since they can exploit spatial relations in gridded training data such as images well, especially considering their relative compactness (typically ranging from 10s to 100s of millions of hyper-parameters). Recurrent neural networks (RNNs) in contrast are the method of choice when predicting dynamic states since their feed-back architecture is designed for sequential data. Research in the field of AI happens either at the application side (i.e., utilizing pre-existing AI methods for novel applications) or at the core (i.e., the design of new architectures and improved numerical optimizers). The application side is fueled by the recent ease of access to AI, facilitated by high-level, relatively easy to use software APIs such as Torch, TensorFlow, Keras, and fast.ai. To exemplify research at the core, we would like to highlight one of the most important ideas of this decade, Generative Adversarial Networks (GANs). The fundamental idea is to set up to deep networks (*generator* and *discriminator*) as adversaries in a game. The generator’s task is to convince the discriminator that it is capable of synthesizing data that is indistinguishable from real data. The discriminator, on the other hand, looks at both synthetic and real-world data and tries to distinguish them. Training the two networks alternatingly results in a generator able to synthesize data convincingly. Uses for GANs include computer-generated art, images of human faces, and so forth.

Natural language processing developed from computational linguistics into an independent field. Beginning with techniques such as Bag of Words (BOW) and skip-gram models to analyze word frequencies and allow for structural comparison of data, the field has evolved rapidly, enabled by new techniques such stemming and lemmatizing to break words into meaningful sub-words, RNN-like architectures are used to exploit the sequential nature of language. Among the most successful recent advances are bidirectional transformer networks such as BERT that are capable of (a) predicting sentences from incomplete beginnings and (b) transform text in one language into an abstract representation that can then be decoded into another language (machine translation).
