# Supplementary material for: Artificial Intelligence in the Fight Against COVID-19: Scoping Review
Source: J Med Internet Res. 2020 Dec 15;22(12):e20756. doi: 10.2196/20756 (PMC7744141; doi:10.2196/20756)
Supplement: Multimedia Appendix 2 [file jmir_v22i12e20756_app2.docx]

Appendix 2: Search Strategy

Database(s): **Ovid MEDLINE(R) and Epub Ahead of Print, In-Process & Other Non-Indexed Citations and Daily**1946 to April 20, 2020
Search Strategy:

| **#** | **Searches** | **Results** |
| --- | --- | --- |
| 1 | exp Coronavirus/ | 11547 |
| 2 | Coronavirus.tw. | 10807 |
| 3 | COVID-19.tw. | 1495 |
| 4 | 2019-nCOV.tw. | 353 |
| 5 | sars-cov-2. tw. | 14 |
| 6 | exp Artificial Intelligence/ | 93805 |
| 7 | Artificial intelligence.tw. | 6139 |
| 8 | Machine learning.tw. | 25035 |
| 9 | Deep learning.tw. | 7397 |
| 10 | Algorithm*.tw. | 245005 |
| 11 | natural language processing.tw. | 2723 |
| 12 | robot*.tw. | 42371 |
| 13 | chatbot*.tw. | 76 |
| 14 | conversational agent*.tw. | 96 |
| 15 | 1 or 2 or 3 or 4 or 5 | 16695 |
| 16 | 6 or 7 or 8 or 9 or 10 or 11 or 12 or 13 or 14 | 358797 |
| 17 | 15 and 16 | 77 |
| 18 | limit 17 to (english language and yr="2019 -Current") | 23 |

Database(s): **Embase**1974 to 2020 April 20
Search Strategy:

| **#** | **Searches** | **Results** |
| --- | --- | --- |
| 1 | exp Coronavirus/ | 11633 |
| 2 | Coronavirus.tw. | 11536 |
| 3 | COVID-19.tw. | 1138 |
| 4 | 2019-nCOV.tw. | 315 |
| 5 | sars-cov-2. tw. | 12 |
| 6 | exp Artificial Intelligence/ | 23548 |
| 7 | Artificial intelligence.tw. | 8183 |
| 8 | Machine learning.tw. | 31214 |
| 9 | Deep learning.tw. | 9308 |
| 10 | Algorithm*.tw. | 312350 |
| 11 | natural language processing.tw. | 3465 |
| 12 | robot*.tw. | 66517 |
| 13 | chatbot*.tw. | 95 |
| 14 | conversational agent*.tw. | 99 |
| 15 | 1 or 2 or 3 or 4 or 5 | 17575 |
| 16 | 6 or 7 or 8 or 9 or 10 or 11 or 12 or 13 or 14 | 416644 |
| 17 | 15 and 16 | 85 |
| 18 | limit 17 to english language | 83 |
| 19 | limit 18 to yr="2019 -Current" | 20 |
| 20 | limit 19 to exclude medline journals | 3 |

Database(s): **APA PsycInfo**1806 to April Week 3 2020
Search Strategy:

| **#** | **Searches** | **Results** |
| --- | --- | --- |
| 1 | exp Coronavirus/ | 0 |
| 2 | Coronavirus.tw. | 65 |
| 3 | COVID-19.tw. | 0 |
| 4 | 2019-nCOV.tw. | 2 |
| 5 | sars-cov-2. tw. | 0 |
| 6 | exp Artificial Intelligence/ | 20538 |
| 7 | Artificial intelligence.tw. | 5033 |
| 8 | Machine learning.tw. | 6108 |
| 9 | Deep learning.tw. | 1175 |
| 10 | Algorithm*.tw. | 32012 |
| 11 | natural language processing.tw. | 1012 |
| 12 | robot*.tw. | 8008 |
| 13 | chatbot*.tw. | 60 |
| 14 | conversational agent*.tw. | 207 |
| 15 | 1 or 2 or 3 or 4 or 5 | 65 |
| 16 | 6 or 7 or 8 or 9 or 10 or 11 or 12 or 13 or 14 | 55549 |
| 17 | 15 and 16 | 0 |

| Database | Search strategy | Hits |
| --- | --- | --- |
| IEEExplore | (("All Metadata":"Artificial intelligence" OR "Machine learning" OR "Deep learning" OR "natural language processing" OR Chatbot* OR Robot*) AND "Abstract":Coronavirus OR "COVID-19" OR "2019-ncov" OR "sars-cov-2"") yr 2019-2020 | 29 |
| ACM Digital library | [[Abstract: coronavirus] OR [Abstract: "covid-19"] OR [Abstract: "sars-cov-2"]] AND [[Abstract: "artificial intelligence"] OR [Abstract: "machine learning"] OR [Abstract: "deep learning"] OR [Abstract: "natural language processing"] OR [Abstract: chatbot*] OR [Abstract: robot*]] AND [Publication Date: (12/01/2019 TO 04/30/2020)] | 0 |
| arXiv | order: -announced_date_first; size: 200; date_range: from 2019-01-01 to 2020-12-31; include_cross_list: True; terms: AND abstract="Artificial intelligence" OR "Machine learning" OR "Deep learning" OR "natural language processing" OR Chatbot* OR Robot*; AND abstract=Coronavirus OR "COVID-19" OR "2019-ncov" | 39 |
| MedRXiv | ("Artificial intelligence" OR "Machine learning" OR "Deep learning") AND (Coronavirus OR "COVID-19" OR "2019-ncov" | 150 |
| BioRXiv | ("Artificial intelligence" OR "Machine learning" OR "Deep learning") AND (Coronavirus OR "COVID-19" OR "2019-ncov OR sars-co") | 62 |
| Scopus | ( ( TITLE-ABS-KEY ( "Artificial Intelligence" ) OR TITLE-ABS-KEY ( "Machine learning" ) OR TITLE-ABS-KEY ( "Deep learning" ) OR TITLE-ABS-KEY ( algorithm* ) OR TITLE-ABS-KEY ( robot* ) OR TITLE-ABS-KEY ( "natural language processing" ) OR TITLE-ABS-KEY ( chatbot* ) OR TITLE-ABS-KEY ( "Conversational agent*" ) ) ) AND ( ( TITLE-ABS-KEY ( coronavirus ) ) OR ( TITLE-ABS-KEY ( covid-19 ) ) OR ( TITLE-ABS-KEY ( 2019-ncov ) ) OR ( TITLE-ABS-KEY ( sars-cov-2 ) ) ) AND ( LIMIT-TO ( PUBYEAR , 2020 ) OR LIMIT-TO ( PUBYEAR , 2019 ) ) | 29 |
| Google Scholar | ("Artificial intelligence" OR "Machine learning" OR "Deep learning" OR "natural language processing" OR Chatbot* OR Robot*) AND (Coronavirus OR "COVID-19" OR "2019-ncov" OR sars-cov-2") | 100 |
