# Supplementary material for: Artificial Intelligence in the Fight Against COVID-19: Scoping Review
Source: J Med Internet Res. 2020 Dec 15;22(12):e20756. doi: 10.2196/20756 (PMC7744141; doi:10.2196/20756)
Supplement: Multimedia Appendix 3 [file jmir_v22i12e20756_app3.docx]

**Appendix 3: Interrater agreement matrices for study selection steps.**

|  | | **Reviewer 1 (AA)** | | |
| --- | --- | --- | --- | --- |
|  |  | **Include** | **Exclude** | **Total** |
| **Reviewer 2 (MA)** | **Include** | **135** | **18** | **153** |
|  | **Exclude** | **13** | **216** | **229** |
|  | **Total** | **148** | **234** | **382** |

**Title and abstract screening**

|  | | **Reviewer 1 (AA)** | | |
| --- | --- | --- | --- | --- |
|  |  | **Include** | **Exclude** | **Total** |
| **Reviewer 2 (MA)** | **Include** | **73** | **1** | **74** |
|  | **Exclude** | **3** | **71** | **74** |
|  | **Total** | **76** | **72** | **148** |

**Full texts reading**
