# Supplementary material for: Artificial Intelligence in the Fight Against COVID-19: Scoping Review
Source: J Med Internet Res. 2020 Dec 15;22(12):e20756. doi: 10.2196/20756 (PMC7744141; doi:10.2196/20756)
Supplement: Multimedia Appendix 4 [file jmir_v22i12e20756_app4.docx]

| **Concept** | **Definition** |
| --- | --- |
| **Study Characteristics** |  |
| Author | The first author of the study. |
| Month of Submission | The month in which the study was submitted. |
| Country of publication | The country where the study was published. |
| Paper status | The current status of the paper (i.e., published or preprint). |
| **AI technique characteristics** |  |
| Purpose/use of AI | What are the applications or uses of AI in COVID-19 pandemic (e.g., diagnosis, drug discovery, projection of cases and deaths)? |
| AI branches | The branches/areas of that were used (e.g., traditional machine learning, deep learning, natural language processing). |
| AI models/ algorithms | The specific AI models or algorithms that were used (e.g., Decision tree, Random forest, Convolutional neural network). |
| Platform | The platform in which the AI technology was implemented (e.g., computers and mobiles). |
| **Dataset** **Characteristics** |  |
| Data sources | Source of data that were used for the development and validation of AI models/ algorithms (e.g., public databases, clinical settings, government sources). |
| Data types | Type of data that were used for the development and validation of AI models/ algorithms (e.g., radiology images, biological data, laboratory data). |
| Dataset size | The total number of data that were used for the development and validation of AI models/ algorithms. |
| Type of validation | How the dataset was split/used to develop and test the proposed models/ algorithms (e.g., Train-test split, K-fold cross-validation, External validation). |
| Proportion of training set | Percentage of the training set of the total dataset. |
| Proportion of validation set | Percentage of validation set of the total dataset. |
| Proportion of test set | Percentage of the test set of the total dataset. |

**Appendix 4: Data extraction form**
