# Supplementary material for: Artificial Intelligence in the Fight Against COVID-19: Scoping Review
Source: J Med Internet Res. 2020 Dec 15;22(12):e20756. doi: 10.2196/20756 (PMC7744141; doi:10.2196/20756)
Supplement: Multimedia Appendix 5 [file jmir_v22i12e20756_app5.docx]

**Appendix 5: Characteristics of the included studies and features of AI techniques used for COVID-19.**

| Author^ID^ | Submission month | Country | Paper status | Purposes/uses of AI techniques | AI branch | AI models/ algorithms | Platform |
| --- | --- | --- | --- | --- | --- | --- | --- |
| Abbas^36^ | April | UK | Preprint | Diagnosis of COVID-19 using X-ray images | DL | CNN | PC |
| Abdelmageed^64^ | March | Sudan | preprint | Vaccine discovery | DL | ANN (unspecified) | PC |
| Al-Qaness^72^ | February | China | Published | Forecasting the epidemic development | DL | ANN (unspecified) | PC |
| Apostolopoulos^37^ | March | Greece | Preprint | Diagnosis of COVID-19 using X-ray images | DL | CNN, TL | PC |
| Bai^95^ | March | China | Preprint | Identifying cases at high risk of progression to severe COVID-19 | DL & ML | MLP, RNN, SVM | PC |
| Barstugan^21^ | March | Turkey | Preprint | Diagnosis of COVID-19 using CT images | ML | SVM | PC |
| Beck^61^ | February | Korea | Preprint | Drug repurposing | DL & NLP | CNN & BERT | PC |
| Bukhari^38^ | March | Pakistan | Preprint | Diagnosis of COVID-19 using X-ray images | DL | CNN | PC |
| Chen J^22^ | March | China | Preprint | Diagnosis of COVID-19 using CT images | DL | CNN | PC |
| Chen X^89^ | April | China | Preprint | Segmentation and quantification of infection regions | DL | CNN | PC |
| Chowdhury^39^ | April | Qatar | Preprint | Diagnosis of COVID-19 using X-ray images | DL | CNN | PC |
| Dandekar^73^ | March | USA | Preprint | Forecasting the epidemic development | DL | ANN (unspecified) | PC |
| DeCapprio^96^ | March | USA | Preprint | Identifying cases at high risk of progression to severe COVID-19 | ML | DT & LoR | PC |
| Dutta^74^ | March | India | Preprint | Forecasting the epidemic development | DL | RNN | PC |
| Fast^68^ | March | USA | Preprint | Protein structure prediction | DL | RNN | PC |
| Feng^49^ | March | China | Preprint | Diagnosis of COVID-19 using laboratory tests | ML | DT, LoR, LASSO, AB | PC |
| Fong-a^75^ | March | China | Preprint | Forecasting the epidemic development | DL | PNN | PC |
| Fong-b^76^ | February | China | Published | Forecasting the epidemic development | DL & ML | DT, LiR, PNN, SVM | PC |
| Fu^23^ | March | China | Preprint | Diagnosis of COVID-19 using CT images | DL | CNN | PC |
| Gaal^90^ | March | Hungary | Preprint | Segmentation and quantification of infection regions | DL | CNN | PC |
| Gao^52^ | February | USA | Preprint | Drug discovery | DL | RNN & DNN | PC |
| Ge^53^ | March | China | Preprint | Drug discovery | DL | DNN | PC |
| Ghoshal^40^ | March | UK | Preprint | Diagnosis of COVID-19 using X-ray images | DL | CNN | PC |
| Gong^97^ | April | China | Preprint | Identifying cases at high risk of progression to severe COVID-19 | ML | LASSO & LoR | PC |
| Gozes^24^ | March | China | Preprint | Diagnosis of COVID-19 using CT images & Segmentation and quantification of infection regions | DL | CNN | PC |
| Guo^86^ | February | China | Preprint | Predicting the potential hosts/reservoirs of 2019-nCov | DL | CNN | PC |
| Hemdan^41^ | March | Egypt | Preprint | Diagnosis of COVID-19 using X-ray images | DL | CNN | PC |
| Heo^69^ | March | USA | Preprint | Protein structure prediction | DL | CNN | PC |
| Hofmarcher^54^ | April | Austria | Preprint | Drug discovery | DL | RNN | PC |
| Hu-a^62^ | March | China | Preprint | Drug repurposing | DL | MDM | PC |
| Hu-b^77^ | March | China | Preprint | Forecasting the epidemic development | DL | AE | PC |
| Hu-c^78^ | March | China | Preprint | Forecasting the epidemic development | DL | AE | PC |
| Huang C^79^ | March | China | Preprint | Forecasting the epidemic development | DL | CNN | PC |
| Huang L^91^ | March | China | Published | Segmentation and quantification of infection regions | DL | CNN | PC |
| Jin C^25^ | March | China | Preprint | Diagnosis of COVID-19 using CT images | DL | CNN | PC |
| Jin S^26^ | March | China | Preprint | Diagnosis of COVID-19 using CT images | DL | CNN | PC |
| Kumar^80^ | April | India | Preprint | Forecasting the epidemic development | ML | ARIMA, VAR, GLM | PC |
| Li L^27^ | March | China | Published | Diagnosis of COVID-19 using CT images | DL | CNN | PC |
| Li M^81^ | April | China | Preprint | Forecasting the epidemic development | ML | EM | PC |
| Lopez-Rincon^50^ | April | Netherlands | Preprint | Diagnosis of COVID-19 using genome sequences | DL | CNN | PC |
| Magar^55^ | March | USA | Preprint | Drug discovery | ML | DT, LoR, MLP, RF, SVM | PC |
| Marini^82^ | April | Switzerland | Preprint | Forecasting the epidemic development | ML | HAM | PC |
| Meng^48^ | March | China | Preprint | Diagnosis of COVID-19 using laboratory tests | ML | LASSO & LoR | PC |
| Mizumoto^83^ | February | Japan | Published | Forecasting the epidemic development | ML | BA | PC |
| Narin^42^ | March | Turkey | Preprint | Diagnosis of COVID-19 using X-ray images | DL | CNN & TL | PC |
| Ong^65^ | March | USA | Preprint | Vaccine discovery | ML | DT, KNN, LoR, SVM, RF | PC |
| Ozturk^43^ | April | Turkey | Published | Diagnosis of COVID-19 using X-ray images | DL | CNN | PC |
| Pandey^102^ | April | India | Preprint | Raising awareness to Water, Sanitation and Hygiene (WASH) | ML & NLP | CBOW, SM, USEL, PS | Mobile |
| Patankar^56^ | March | UK | Preprint | Drug discovery | DL | RNN & AE | PC |
| Pirouz^84^ | March | China | Published | Forecasting the epidemic development | DL | PNN | PC |
| Pourhomayoun^98^ | April | USA | Preprint | Patient outcome prediction (mortality rate) | DL & ML | ANN, DT, KNN, LoR, RF, SVM | PC |
| Qi^101^ | March | China | Preprint | Patient outcome prediction (length of hospital stay) | ML | LoR & RF | PC |
| Qiang^87^ | March | China | Published | Predicting the potential hosts/reservoirs of 2019-nCov | ML | RF | PC |
| Qiao^70^ | March | Canada | Preprint | Protein structure prediction | DL | CNN & RNN | PC |
| Rahman^66^ | March | Bangladesh | Preprint | Vaccine discovery | DL | ANN (unspecified) | PC |
| Randhawa^88^ | February | Canada | Preprint | Predicting the potential hosts/reservoirs of 2019-nCov | ML | KNN, LDA, SVM | PC |
| Saçar demirci^71^ | March | Turkey | Preprint | Protein structure prediction | ML | DT, NB, RF | PC |
| Sarkar B^67^ | March | Bangladesh | Preprint | Vaccine discovery | ML | SVM | PC |
| Sarkar J^100^ | March | India | Preprint | Patient outcome prediction (risk factors associated with mortality) | ML | RF | PC |
| Sethy^44^ | April | India | Preprint | Diagnosis of COVID-19 using X-ray images | DL & ML | CNN, SVM, TL | PC |
| Shan^92^ | March | China | Preprint | Segmentation and quantification of infection regions | DL & ML | CNN | PC |
| Shi^28^ | March | China | Preprint | Diagnosis of COVID-19 using CT images | DL & ML | CNN, RF, LoR, SVM | PC |
| Tang B^57^ | March | China | Preprint | Drug discovery | DL | ADQN | PC |
| Tang Z^93^ | March | China | Preprint | Severity assessment | ML | RF | PC |
| Tiwari^85^ | April | India | Preprint | Forecasting the epidemic development | ML | TSF | PC |
| Ton^58^ | March | Canada | Published | Drug discovery | DL | DNN | PC |
| Ucar^45^ | April | Turkey | Published | Diagnosis of COVID-19 using X-ray images | DL | CNN | PC |
| Wang L^46^ | March | Canada | Preprint | Diagnosis of COVID-19 using X-ray images | DL | CNN | PC |
| Wang Shuai^29^ | April | China | Preprint | Diagnosis of COVID-19 using CT images | DL | CNN | PC |
| Wang Shuo^30^ | March | China | Preprint | Diagnosis of COVID-19 using CT images & Identifying cases at high risk of progression to severe COVID-19 | DL | CNN | PC |
| Wang Y^51^ | February | China | Preprint | Identification of suspected COVID-19 based on respiratory patterns | DL | RNN | PC |
| Wang Z^63^ | February | China | Preprint | Predicting the safety of Traditional Chinese Medicine | DL | ANN (unspecified) | PC |
| Xu^31^ | February | China | Preprint | Diagnosis of COVID-19 using CT images | DL | CNN | PC |
| Yan^99^ | March | China | Preprint | Patient outcome prediction (mortality rate) | ML | DT | PC |
| Ying^32^ | February | China | Preprint | Diagnosis of COVID-19 using CT images | DL | CNN | PC |
| Yu^94^ | March | China | Preprint | Severity assessment | ML | DT | PC |
| Zhang H^59^ | February | China | Preprint | Drug discovery | DL | CNN | PC |
| Zhang J^47^ | March | China | Preprint | Diagnosis of COVID-19 using X-ray images | DL | CNN | PC |
| Zhao^33^ | March | USA | Preprint | Diagnosis of COVID-19 using CT images | DL | CNN | PC |
| Zhavoronkov^60^ | February | Honk Kong | Preprint | Drug discovery | DL & ML | AE, GAN, GA, LM, RL | PC |
| Zheng^34^ | March | China | Preprint | Diagnosis of COVID-19 using CT images | DL | CNN | PC |
| Zhou^35^ | March | China | Preprint | Diagnosis of COVID-19 using CT images | DL | CNN & TL | PC |
| Abbreviations | **AB**: AdaBoost; **ADQN**: Advance Deep Q-learning network; **AE**: Auto-encoders; **ANN**: Artificial Neural Network (unspecified); **ARIMA**: Auto-Regressive Integrated Moving Average Model; **BA**: Bayesian analysis; **BERT**: Bidirectional Encoder Representations from Transformers; **CBOW**: Continuous Bag of Words; **CNN**: Convolutional neural network; **DL**: Deep Learning; **DNN**: Deep neural network; **DT**: Decision tree; **EM**: Eureqa Modelling; **GA**: Genetic algorithm; **GAN**: Generative adversarial network; **GLM**: Generalized Logistic growth Model; **HAM**: Holistic Agent-based Model; **KNN**: K-Nearest Neighbors; **LASSO**: Least Absolute Shrinkage and Selection Operator; **LDA**: Linear Discriminant Analysis; **LiR**: Linear Regression; **LM**: Language model; **LoR**: Logistic Regression; **MDM**: Multi-task deep model; **DL**: Machine Learning; **MLP**: Multilayer perceptron; **NB**: Naive Bayes; **NLP**: Natural Language Processing; **PNN**: Polynomial Neural Network; **PS**: Porter Stemming; **RF**: Random Force; **RL**: Reinforcement learning; **RNN**: Recurrent Neural Network; **SM**: Skip-gram model; **SVM**: Support Vector Machine; **TL**: Transfer learning; **TSF**: Time Series Forecasting; **USEL**: Universal-sentence-encoder-large; **VAR**: Vector Auto Average. | | | | | | |
