# Supplementary material for: Artificial Intelligence in the Fight Against COVID-19: Scoping Review
Source: J Med Internet Res. 2020 Dec 15;22(12):e20756. doi: 10.2196/20756 (PMC7744141; doi:10.2196/20756)
Supplement: Multimedia Appendix 6 [file jmir_v22i12e20756_app6.docx]

**Appendix 6: Features of datasets used for development and validation of AI models**

| Author^ID^ | Data source | Data type | Dataset size | Type of validation | % of training set | % of validation set | % of test set |
| --- | --- | --- | --- | --- | --- | --- | --- |
| Abbas^36^ | Public database | Radiology images | 196 | Training-test split | 70% | N/A | 30% |
| Abdelmageed^64^ | Public database | Biological data | N/A | N/A | N/A | N/A | N/A |
| Al-Qaness^72^ | Public database | Epidemiological Data | N/A | Training-test split | 75% | N/A | 25% |
| Apostolopoulos^37^ | Public database & Literature | Radiology images | 1427 | 10-fold cross-validation | 90% | N/A | 10% |
| Bai^95^ | Clinical setting | Radiology images, clinical data, and laboratory data | 133 | 5-fold cross-validation | 80% | N/A | 20% |
| Barstugan^21^ | Public database | Radiology images | 150 | 2-fold, 5-fold and 10-fold cross-validations | 50%, 80%, 90% | N/A | 10%,20%, 50% |
| Beck^61^ | Public database | Biological data | N/A | N/A | N/A | N/A | N/A |
| Bukhari^38^ | Public database | Radiology images | 278 | Training-test split | 80% | N/A | 20% |
| Chen J^22^ | Clinical setting | Radiology images, clinical data, and laboratory data | 24907 | Training-test split | 4% | 40% | 56% |
| Chen X^89^ | Public database | Radiology images | 110 | 10-fold cross-validation | 90% | N/A | 10% |
| Chowdhury^39^ | Public database & Literature | Radiology images | 8482 | 5-fold cross-validation | 96% | N/A | 4% |
| Dandekar^73^ | Government sources | Epidemiological Data | N/A | N/A | N/A | N/A | N/A |
| DeCapprio^96^ | Government sources | Clinical data & Demographic data | 1851519 | Training-test split | 80% | N/A | 20% |
| Dutta^74^ | Public database | Epidemiological Data | N/A | N/A | N/A | N/A | N/A |
| Fast^68^ | Public database | Biological data | N/A | N/A | N/A | N/A | N/A |
| Feng^49^ | Clinical setting | Clinical data, demographic data, laboratory data | 132 | 10-fold cross-validation | 80% | N/A | 20% |
| Fong-a^75^ | Government sources | Epidemiological Data | N/A | N/A | N/A | N/A | N/A |
| Fong-b^76^ | Government sources | Epidemiological Data | N/A | N/A | N/A | N/A | N/A |
| Fu^23^ | Clinical setting | Radiology images | 89628 | External validation | 50% | 17% | 33% |
| Gaal^90^ | Public database | Radiology images | 1047 | Training-test split | N/A | N/A | N/A |
| Gao^52^ | Public database | Biological data | N/A | N/A | N/A | N/A | N/A |
| Ge^53^ | Public database | Biological data | N/A | N/A | N/A | N/A | N/A |
| Ghoshal^40^ | Public database | Radiology images | 5941 | Training-test split | 80% | N/A | 20% |
| Gong^97^ | Clinical setting | Clinical data, demographic data, laboratory data | 372 | Training-test split | 51% | 44% | 5% |
| Gozes^24^ | Public database & Clinical setting | Radiology images | 207 | External validation | 24% | N/A | 76% |
| Guo^86^ | Public database | Biological data | N/A | N/A | N/A | N/A | N/A |
| Hemdan^41^ | Public database | Radiology images | 50 | Training-test split | 80% | N/A | 20% |
| Heo^69^ | Public database | Biological data | N/A | N/A | N/A | N/A | N/A |
| Hofmarcher^54^ | Public database | Biological data | N/A | N/A | N/A | N/A | N/A |
| Hu-a^62^ | Public database | Biological data | N/A | N/A | N/A | N/A | N/A |
| Hu-b^77^ | Public database | Epidemiological Data | N/A | N/A | N/A | N/A | N/A |
| Hu-c^78^ | Public database | Epidemiological Data | N/A | N/A | N/A | N/A | N/A |
| Huang C^79^ | Public database | Epidemiological Data | N/A | External validation | N/A | N/A | N/A |
| Huang L^91^ | Clinical setting | Radiology images | 842 | Training-test split | 91% | N/A | 9% |
| Jin C^25^ | Public database & Clinical setting | Radiology images | 1671 | External validation | 19% | 6% | 75% |
| Jin S^26^ | Clinical setting | Radiology images | 1418 | Training-test split | 80% | N/A | 20% |
| Kumar^80^ | Public database | Epidemiological Data | N/A | N/A | N/A | N/A | N/A |
| Li L^27^ | Clinical setting | Radiology images | 4356 | Training-test split | 90% | N/A | 10% |
| Li M^81^ | Public database & Government sources | Epidemiological Data | N/A | N/A | N/A | N/A | N/A |
| Lopez-Rincon^50^ | Public database & Government sources | Biological data | 553 | 10-fold cross-validation | 80% | 10% | 10% |
| Magar^55^ | Public database | Biological data | 1933 | 5-fold cross-validation | 80% | N/A | 20% |
| Marini^82^ | Public database | Epidemiological Data | N/A | N/A | N/A | N/A | N/A |
| Meng^48^ | Clinical setting | Clinical data & laboratory data | 620 | External validation | 70% | N/A | 30% |
| Mizumoto^83^ | Government sources | Laboratory data | 634 | N/A | N/A | N/A | N/A |
| Narin^42^ | Public database | Radiology images | 100 | 5-fold cross-validation | 80% | N/A | 20% |
| Ong^65^ | Public database | Biological data | N/A | 5-fold cross-validation | N/A | N/A | N/A |
| Ozturk^43^ | Public database | Radiology images | 1125 | 5-fold cross-validation | 80% | N/A | 20% |
| Pandey^102^ | Public database & News websites | Guidelines & news | N/A | N/A | N/A | N/A | N/A |
| Patankar^56^ | Public database | Biological data | 310000 | Training-test split | 75% | N/A | 25% |
| Pirouz^84^ | Public database | Epidemiological Data | 64786 | Training-test split | 75% | N/A | 25% |
| Pourhomayoun^98^ | Literature, Government sources, News websites | Clinical data & Demographic data | 117000 | 10-fold cross-validation | 90% | N/A | 10% |
| Qi^101^ | Clinical setting | Radiology images | 31 | 5-fold cross-validation | 80% | N/A | 20% |
| Qiang^87^ | Government sources | Biological data | 2666 | 10-fold cross-validation | 90% | N/A | 10% |
| Qiao^70^ | Public database | Biological data | N/A | N/A | N/A | N/A | N/A |
| Rahman^66^ | Public database | Biological data | 250 | N/A | N/A | N/A | N/A |
| Randhawa^88^ | Public database | Biological data | N/A | 10-fold cross-validation | 90% | N/A | 10% |
| Saçar demirci^71^ | Public database | Biological data | N/A | N/A | N/A | N/A | N/A |
| Sarkar B^67^ | Public database | Biological data | N/A | N/A | N/A | N/A | N/A |
| Sarkar J^100^ | Public database | Clinical data | 430 | Training-test split | 70% | N/A | 30% |
| Sethy^44^ | Public database | Radiology images | 183 | Training-test split | 60% | 20% | 20% |
| Shan^92^ | Clinical setting | Radiology images | 549 | External validation | 45% | N/A | 55% |
| Shi^28^ | Clinical setting | Radiology images | 2685 | 5-fold cross-validation | 80% | N/A | 20% |
| Tang B^57^ | Public database | Biological data | N/A | N/A | N/A | N/A | N/A |
| Tang Z^93^ | Clinical setting | Radiology images | 176 | 3-fold cross-validation | 65% | N/A | 35% |
| Tiwari^85^ | Public database | Epidemiological Data | N/A | Training-test split | 70% | N/A | 30% |
| Ton^58^ | Public database & Literature | Biological data | 3000000 | N/A | N/A | N/A | N/A |
| Ucar^45^ | Public database | Radiology images | 5949 | Training-test split | 80% | 10% | 10% |
| Wang L^46^ | Public database | Radiology images | N/A | Training-test split | N/A | N/A | N/A |
| Wang Shuai^29^ | Clinical setting | Radiology images | 453 | External validation | 52% | N/A | 48% |
| Wang Shuo^30^ | Clinical setting | Radiology images | 1266 | External validation | 56% | N/A | 44% |
| Wang Y^51^ | Participants | Radiology images | 120605 | External validation | 99.50% | N/A | 0.50% |
| Wang Z^63^ | Literature | Clinical data | N/A | 10-fold cross-validation | 90% | N/A | 10% |
| Xu^31^ | Clinical setting | Radiology images | 618 | Training-test split | 85% | N/A | 15% |
| Yan^99^ | Clinical setting | Clinical data, demographic data, laboratory data, epidemiological data | 404 | External validation | 66% | 27% | 7% |
| Ying^32^ | Clinical setting | Radiology images | 1990 | Training-test split | 60% | 10% | 30% |
| Yu^94^ | Clinical setting | Clinical data, laboratory data, epidemiological data | 105 | N/A | N/A | N/A | N/A |
| Zhang H^59^ | Public database | Biological data | N/A | N/A | N/A | N/A | N/A |
| Zhang J^47^ | Public database | Radiology images | 1531 | Training-test split | 93% | N/A | 7% |
| Zhao^33^ | Public database & Literature | Radiology images | 471 | Training-test split | 60% | 15% | 15% |
| Zhavoronkov^60^ | Public database & Clinical setting | Biological data | N/A | N/A | N/A | N/A | N/A |
| Zheng^34^ | Clinical setting | Radiology images | 630 | Training-test split | 79% | N/A | 21% |
| Zhou^35^ | Clinical setting | Radiology images | 4474 | Training-test split & External validation | 63% | 7% | 30% |
| Abbreviations | N/A: not available |  |  |  |  |  |  |
